# RBFOX3 regulates Claudin-1 expression in human lung tissue via attenuation of proteasomal degradation

**DOI:** 10.1042/BSR20160623

**Published:** 2017-02-23

**Authors:** Yong-Eun Kim, Sunkyung Choi, Jong Ok Kim, Kee K. Kim

**Affiliations:** 1 Department of Biochemistry, Chungnam National University, Daejeon 34134, Republic of Korea; 2 Department of Pathology, Daejeon Saint Mary’s Hospital, The Catholic University of Korea, Daejeon 34943, Republic of Korea

**Keywords:** lung tissue, protein stability, RBFOX3, tight junctions, ubiquitin-proteasomal degradation

## Abstract

RBFOX3, a nuclear RNA-binding protein, is well known as a regulator of alternative pre-mRNA splicing during neuronal development. However, other functions of RBFOX3 are poorly understood. Here, we investigated the function of RBFOX3 in the cytoplasm with respect to regulation of Claudin-1 expression. In human lung tissue, Claudin-1 is higher in RBFOX3-positive cells than in RBFOX3-negative cells. Immunostaining and mRNA quantification revealed that protein levels, but not mRNA levels, of Claudin-1 are increased by RBFOX3. In addition, cycloheximide treatment of human lung cancer cells revealed that RBFOX3 increases the stability of Claudin-1 through attenuation of its ubiquitination. Our study provides insights into the molecular mechanisms by which RBFOX3 regulates Claudin-1 expression in human lung tissue.

## Introduction

RNA-binding proteins play important roles in regulation of gene expression by affecting mRNA stability, translation, and miRNA biogenesis, and through formation of splicing networks. The RBFOX family is a well-known group of RNA-binding proteins that is involved in tissue-specific alternative splicing. There are three RBFOX family members in mammals RBFOX1, RBFOX2, and RBFOX3. RBFOX1 is mainly expressed in neurons, heart and skeletal muscles, whereas RBFOX2 is expressed ubiquitously in various tissues as well as in embryonic stem cells. On the contrary, RBFOX3 is predominantly expressed in the neural tissue [1–3]. RBFOX1 has critical roles in diverse developmental processes, including germ cell differentiation [4], whereas RBFOX2 is found to be a fundamental regulator of epithelial–mesenchymal transition (EMT)-mediated alternative splicing, which promotes cellular invasion [5].

RBFOX3 is known as a regulator of miRNA biogenesis [6]. Interestingly, although RBFOX3 is predominantly expressed in most neuronal cell types, recent reports revealed that RBFOX3 is also frequently expressed in epithelial neuroendocrine carcinoma and non-small cell lung cancer (NSCLC) [7,8]. In addition, even though RBFOX3 is principally nuclear protein, it was also detected in the cytoplasm of many neuronal cell types and tumor tissues, especially NSCLC tissue [8–10]. Although most of the studies on RBFOX3 have focused on splicing regulation in neural tissue, the roles of RBFOX3 may not be limited to splicing regulation. The mechanism underlying the effect of RBFOX3-mediated cytoplasmic functions on gene expression in the lung tissue is largely unknown. RBFOX3 regulate numerous aspects of gene expression; hence, investigation of the cytoplasmic function of RBFOX3 is important for understanding its physiological functions in the lung tissue.

Epithelial cells maintain cellular morphology and polarity by forming tight junctions with neighboring cells. Claudins are tight junction proteins that play critical roles in preserving epithelial integrity. The Claudin family of proteins is composed of at least 24 members; these proteins have four transmembrane domains, two extracellular loops and one C-terminal intracellular tail [11]. *CLDN1*, which encodes Claudin-1, is expressed in a tissue-specific manner and is involved in the proliferation, invasive ability, and metastasis of tumor cells. The expression of Claudin-1 varies among tumor tissues—high levels have been observed in lung squamous cell, colon and liver carcinoma, and low levels in breast carcinoma [12]. The mRNA level of *CLDN1* was epigenetically increased by regulation of histone deacetylase activity in colon tumor [13]. Moreover, *CLDN1* expression is reduced by the β-catenin/TGF signaling pathway in EMT [14]. However, the mechanisms that regulate Claudin-1 expression and its stability in human lung tissue are unknown. Recently, we showed that siRNA-mediated depletion of RBFOX3 or TGF-β-mediated reduction in RBFOX3 reduces Claudin-1 levels. These results suggest that RBFOX3 is associated with regulation of *CLDN1* expression [10].

In the present study, we investigated how RBFOX3 regulates *CLDN1* expression. We found that *CLDN1* is expressed highly in RBFOX3-positive cells of human lung tissues where localization of RBFOX3 was specifically cytoplasmic. Furthermore, stability of Claudin-1 is increased by RBFOX3 through inhibition of its ubiquitination. These results unveil a novel function of RBFOX3 as a regulator of Claudin-1.

## Experimental

### Cell culture reagents

A549 human lung adenocarcinoma cells were purchased from American Tissue Culture Collection (Manassas, VA, U.S.A.) and maintained in Dulbecco’s modified Eagle medium supplemented with 10% heat-inactivated fetal bovine serum (GIBCO, NY, U.S.A.) at 37°C in a humid 5% CO_2_ atmosphere. The proteasome inhibitor MG132 (carbobenzoxy-L-leucyl-L-leucyl-L-leucinal) and protein synthesis inhibitor cycloheximide (CHX) were purchased from Calbiochem (Darmstadt, Germany) and Sigma–Aldrich (MO, U.S.A.) respectively. Protein G dynabeads for immunoprecipitation was purchased from ThermoFisher Scientific (MA, U.S.A.). D-luciferin and coelenterazine-h were purchased from Promega (WI, U.S.A.).

### Plasmids and transfection reagents

The expression construct encoding the N-terminal Myc-tagged mouse Rbfox3 in the pCS3+MT vector has been described earlier [2]. Expression constructs encoding Myc-tagged mouse Rbfox1 and Rbfox2 in pCS3+MT vector were also described previously as A016 and F411 respectively [15]. For the luciferase assay, the plasmid pNF-κB-Luc expressing firefly luciferase and the control reporter plasmid pRL expressing *Renilla* luciferase were purchased from Stratagene (CA, U.S.A.) and Promega (WI, U.S.A.) respectively. All transfection experiments were performed using Lipofectamine 2000 reagents (ThermoFisher Scientific, MA, U.S.A.) according to the manufacturer’s instruction.

### RNA preparation and quantitative reverse transcription-PCR

Total RNA was isolated from cultured cells using GeneAll Hybrid-R RNA purification kit (GENEALL, Korea). Reverse transcription PCR (RT-PCR) was conducted with M-MLV reverse transcriptase (Promega, WI, U.S.A.) using random hexamers. Quantitative PCR (qPCR) was performed using Prime Q-Mastermix (GENETBIO, Korea) and SYBER-green fluorescent dye. The following primers were used: Claudin-1, 5′-GCA GAT CCA GTG CAA AGT CT-3′ and 5′-CAT ACA CTT CAT GCC AAC GG-3′; β-actin, 5′-TAC CCC ACA CTG TGC CCA TCT ACG A-3′ and 5′-CAG CGG AAC CGC TCA TTG CCA ATG-3′. Quantitative RT-PCR (qRT-PCR) was performed in the AriaMx Real-Time PCR system (Agilent Technologies) using the following conditions: initial denaturation at 95°C for 5 min, followed by 40 cycles of 95°C for 20 s, 58°C for 20 s, and 72°C for 20 s. The specificity of PCR products was confirmed by melting curve analysis of qRT-PCR and gel electrophoresis of the PCR products.

### Cell lysate preparation, immunoprecipitation, and immunoblot analysis

A549 cells were extracted using M-PER protein extraction reagent (ThermoFisher Scientific, MA, U.S.A.), which was supplemented with a complete protease inhibitor cocktail (Roche, Basel, Switzerland). Protein concentration was determined using the Qubit protein assay kit (ThermoFisher Scientific, MA, U.S.A.). Thirty microgram lysate per sample was loaded and separated on SDS/PAGE (12% gel). For immunoprecipitation of Claudin-1, lysates of A549 cells were prepared in IP lysis buffer (25 mM Tris/HCl pH7.5, 150 mM NaCl, 1 mM EDTA, 1% NP-40, and 5% glycerol) containing complete protease inhibitor cocktail. The supernatant was incubated with anti-Claudin-1 antibody or IgG for 3 h at 4°C; immune complexes were isolated by incubating with protein G dynabeads. The collected immune complexes were separated by SDS/PAGE (12% gel) and transferred to a PVDF membrane. Immunoblotting was performed as described previously [16]. The antibodies utilized in this immunoblot analysis were anti-Claudin-1 (Cell Signaling Technologies, MA, U.S.A.), anti-ubiquitin (Cell Signaling Technologies, MA, U.S.A.), anti-Myc (ThermoFisher Scientific, MA, U.S.A.), anti-RBFOX3 (EMD Millipore, Darmstadt, Germany), and anti-GAPDH (Meridian Life Science, TN, U.S.A.).

### Immunofluorescence microscopy

A549 cells were fixed with 4% paraformaldehyde for 10 min and permeabilized with 0.5% Triton X-100 in PBS for 15 min. The fixed cells were blocked with BSA, followed by overnight incubation with anti-Claudin-1 and anti-Myc antibodies. Frozen sections of human normal lung tissue were purchased from BioChain (CA, U.S.A.). The sections were stained with anti-Claudin-1 and anti-RBFOX3 antibodies. Alexa Fluor 488- and Alexa Fluor 532-conjugated goat antibodies against mouse and rabbit IgG (ThermoFisher Scientific, MA, U.S.A.), respectively, were used as secondary antibodies. Nuclei were stained with DAPI (ThermoFisher Scientific, MA, U.S.A.). Images were captured by an LSM 510 Live Configuration Vario Two VRGB confocal laser-scanning microscope (Carl Zeiss, Oberkchen, Germany).

### Luciferase reporter assay

A549 cells were transfected with the plasmid expressing Myc-tagged RBFOX3 and the firefly luciferase reporter plasmid containing multimerized NF-κB response elements. Constitutively expressed *Renilla* luciferase plasmid was co-transfected for normalization of transfection efficiency. After 48 h, transfected cells were washed with PBS and lysed with passive lysis buffer (Promega, WI, U.S.A.). Firefly and *Renilla* luciferase activities were measured using an LB 953 Autolumat (EG&G Berthold, Nashua, NH, U.S.A.) by sequentially adding luciferin and coelenterazine-h, respectively.

## Results

### CLDN1 is highly expressed in RBFOX3-positive cells in the human lung tissue

**Figure 1:**
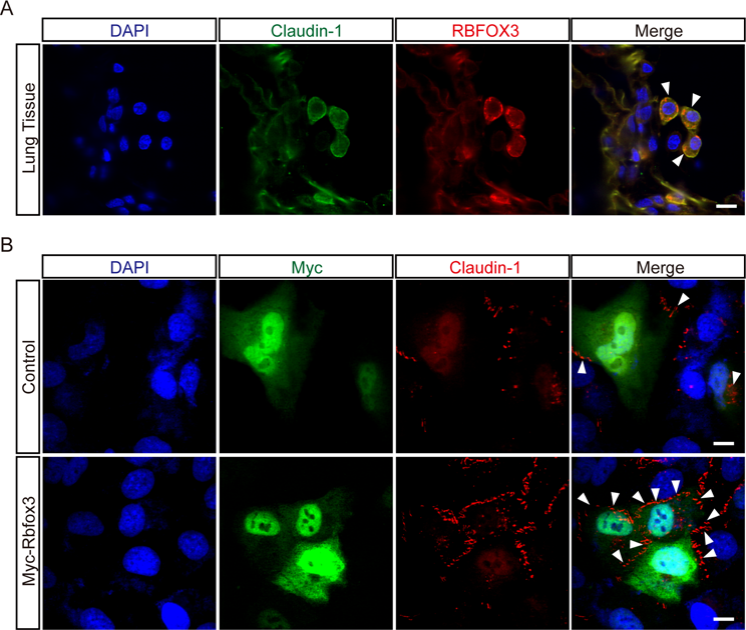
*CLDN1* is highly expressed in RBFOX3-positive cells (**A**) Human lung tissue was immunostained with anti-Claudin-1 (green) and anti-RBFOX3 (red) antibodies. The arrowheads indicate the double positive cells of Claudin-1 and RBFOX3. (**B**) A549 cells were transfected with plasmid harboring Myc-tagged RBFOX3 or empty plasmid. Forty-eight hours after transfection, the cells were immunostained with anti-Myc (green) and anti-Claudin-1 (red) antibodies. Arrowheads indicate cells double positive for Claudin-1 and RBFOX3. DAPI (blue) was used to stain nuclear. Scale bars indicate 10 μm.

Our observation that protein level of Claudin-1 was decreased in RBFOX3-depleted A549 human lung adenocarcinoma cells prompted us to ask whether expression of *RBFOX3* correlates with that of *CLDN1* in human lung tissue. *CLDN1* is known to be highly expressed in lung squamous cell carcinoma [12]. To examine whether *CLDN1* is expressed in normal human lung tissue, we performed immunofluorescence microscopy using anti-Claudin-1 and anti-RBFOX3 antibodies. As Claudin-1 is a well-known tight junction protein, we observed cytoplasmic staining pattern of Claudin-1, whose intensity varied among cells (Figure 1A). Although translocalization of Claudin-1 from the membrane to the cytoplasm by activation of protein kinase C in epithelial cells is known, the molecular function of cytoplasmic Claudin-1 has not been determined yet. [17]. Unlike the nuclear localization of RBFOX3 in neuronal cells, punctate staining was observed for RBFOX3 in the cytoplasm of lung tissue. This result suggests that the function of RBFOX3 in the lung tissue differs from its splicing regulation in neuronal cells. Interestingly, *CLDN1* was strongly expressed in RBFOX3-positive cells, suggesting that the cytoplasmic function of RBFOX3 could be associated with *CLDN1* expression. To investigate whether RBFOX3 directly regulates *CLDN1* expression, we examined the levels of Claudin-1 in A549 cells that were overexpressing RBFOX3. Although majority of the overexpressed RBFOX3 is nuclear, a marginal amount remained in the cytoplasm (Figure 1B). However, we could not observe any co-staining between RBFOX3 and Claudin-1, suggesting that RBFOX3 may not be a direct regulator of *CLDN1* expression. Importantly, RBFOX3-expressing cells displayed high level of Claudin-1, thereby implying that *CLDN1* expression positively correlated with that of *RBFOX3* in lung tissue (Figure 1B).

### RBFOX3 increases *CLDN1* expression post-transcriptionally

**Figure 2:**
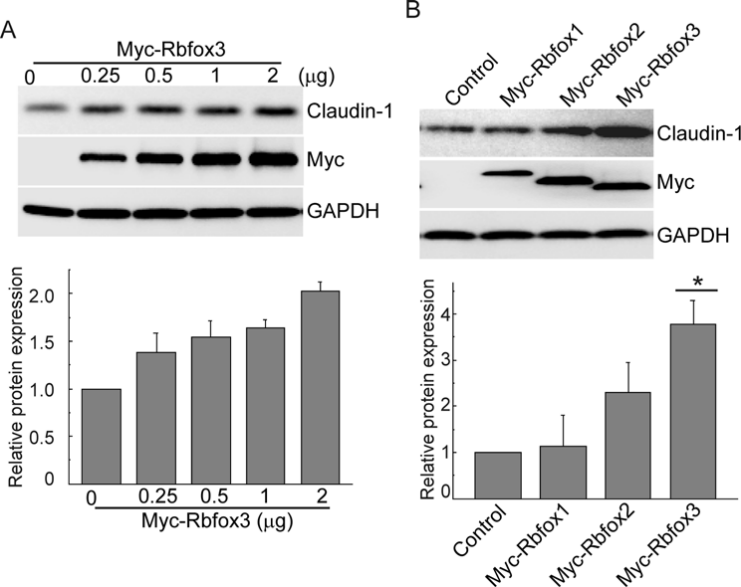
RBFOX3 increases *CLDN1* expression (**A**) A549 cells were transfected with increasing amounts of the plasmid harboring Myc-tagged RBFOX3. Forty-eight hours after transfection, the cells were subjected to immunoblot analysis with the indicated antibodies. The graph presents the trend in normalized Claudin-1 level (relative to GAPDH) as a function of increasing amounts of the plasmid harboring Myc-RBFOX3. (**B**) A549 cells were transfected with plasmids harboring Myc-tagged RBFOX1, 2, or 3. Forty-eight hours after transfection, the cells were subjected to immunoblot analysis with the indicated antibodies. The graph presents normalized Claudin-1 level (relative to GAPDH) in the different types of transfectants. Data are presented as mean values ± S.D. (*n*=3), *P<0.05 (Student’s *t-*test).

Although *CLDN1* expression may not regulated directly by RBFOX3, levels of RBFOX3 strongly correlated with that of Claudin-1. Exogenous RBFOX3 expression increased Claudin-1 levels (Figure 2A). RBFOX family proteins exist as diverse isoforms, which differ in their intracellular localization. Therefore, we next investigated whether other RBFOX isoforms are involved in modulating *CLDN1* expression. Although the levels of the three RBFOX isoforms were comparable, RBFOX1 showed lesser effect on *CLDN1* expression than RBFOX3 did (Figure 2B). We postulated that the varying extents of RBFOX-mediated modulation of *CLDN1* expression are due to differences in their intracellular localization.

**Figure 3:**
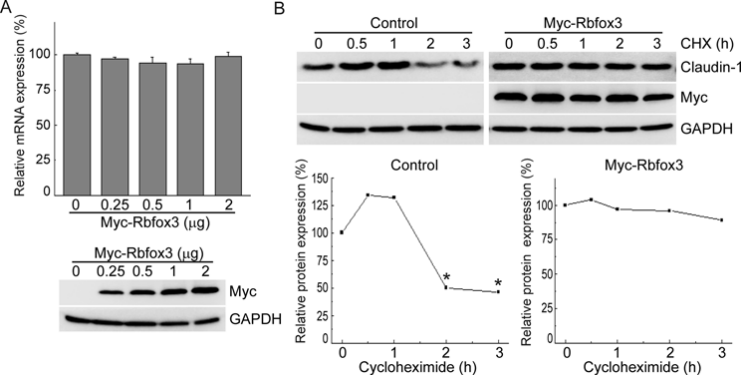
RBFOX3 increases *CLDN1* expression post-transcriptionally (**A**) A549 cells were transfected with increasing amounts of the plasmid harboring Myc-tagged RBFOX3. Forty-eight hours after transfection, mRNA level of *CLDN1* was measured by quantitative RT-PCR. (**B**) A549 cells were transfected with 2 μg of Myc-tagged RBFOX3. Forty-eight hours after transfection, the cells were treated with 50 μg/ml CHX for the indicated times. The cells were subjected to immunoblot analysis with the indicated antibodies. Band intensity of Claudin-1 was normalized to that of GAPDH. The graph presents the relative density with respect to the zero-time point; *P<0.05 (Student’s *t-*test).

Next, to investigate the molecular mechanism of RBFOX3-mediated modulation of *CLDN1* expression, we first examined its effect on mRNA levels of *CLDN1*. Interestingly, we observed no significant change in the mRNA levels of *CLDN1* upon RBFOX3 overexpression, suggesting that the latter does not affect transcription of *CLDN1* (Figure 3A). Our observation that RBFOX3 is cytoplasmic in human lung tissue prompted us to investigate whether RBFOX3 affects stability of Claudin-1 in the cytoplasm. Toward this goal, we blocked *de novo* protein synthesis by treating A549 cells overexpressing RBFOX3 with CHX and analyzed the levels of Claudin-1 at different time points of the treatment. In cells that overexpressed RBFOX3, 98% of Claudin-1 persisted 2 h after CHX treatment (Figure 3B). In contrast, Claudin-1 levels halved by 2 h in the absence of RBFOX3, suggesting that RBFOX3 regulates Claudin-1 expression by modulating protein stability.

### RBFOX3 regulates ubiquitination of Claudin-1

**Figure 4:**
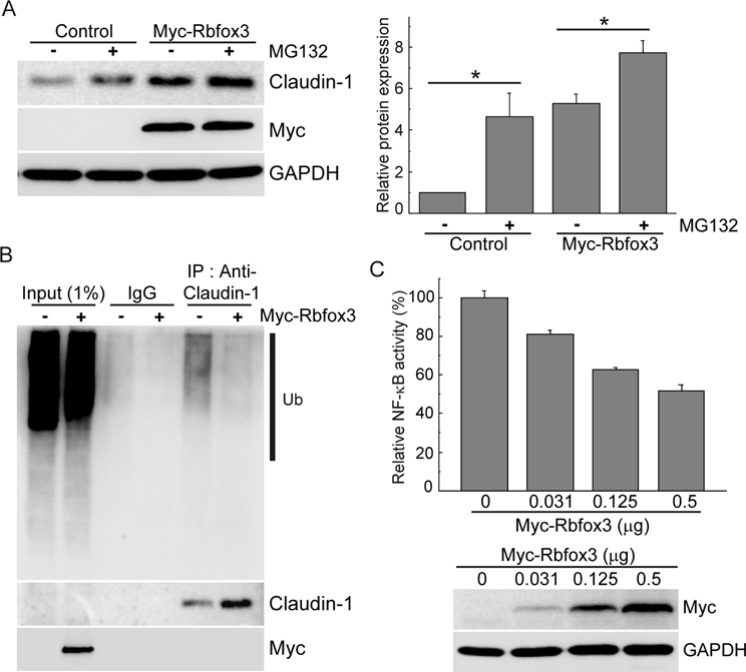
RBFOX3 regulates ubiquitination of Claudin-1 (**A**) A549 cells were transfected with the plasmid harboring Myc-tagged RBFOX3. Thirty-six hours after transfection, cells were treated with 1 μM MG132 for 12 h. The cells were subjected to immunoblot analysis with the indicated antibodies. The graph presents the relative levels of Claudin-1. Data are presented as mean values ± S.D. (*n*=3), *P<0.05 (Student’s *t-*test). (**B**) A549 cells were transfected with the plasmid harboring Myc-tagged RBFOX3. Forty-eight hours after transfection, cells were treated with 20 μM MG132 for 2 h. Cell lysates were subjected to immunoprecipitation with anti-Claudin-1 antibody, and the precipitates were subjected to immunoblot analysis by the indicated antibodies. Ub, Ubiquitin. (**C**) A549 cells were transfected with the luciferase reporter plasmid containing multimerized NF-κB-responsive element together with increasing doses of the plasmid harboring Myc-tagged RBFOX3. Luciferase activity is presented relative to that in control cells after normalization with *Renilla* luciferase. Data are presented as mean values ± S.D. (*n*=3). Immunoblot analysis using anti-Myc antibody (lower panel) shows amounts of the expressed proteins.

In tight junctions of MDCK cells, Claudin-1 is ubiquitinated by the E3 ubiquitin ligase LNX1p80, following which Claudin-1 levels decrease through endocytosis and lysosomal degradation [18,19]. However, the mechanism of Claudin-1 degradation has not been investigated in lung tissue. Therefore, to determine whether Claudin-1 is degraded via the proteasome pathway, we analyzed the effect of proteasome inhibitor MG132 on Claudin-1 levels. Claudin-1 level was increased by MG132 treatment in both the control and RBFOX3 overexpressing cells, suggesting that Claudin-1 is degraded by the proteasome pathway (Figure 4A). Ubiquitination is an essential step in degradation through the ubiquitin–proteasome pathway (UPP) [20]. To determine whether RBFOX3 is involved in ubiquitination of Claudin-1, we monitored the ubiquitination status of Claudin-1 in MG132-treated A549 cells. A549 cells were transfected with either an empty vector or a Myc-tagged RBFOX3 plasmid. Forty-eight hours after transfection, the cells were treated with high concentration of MG132 (20 μM) for 2 h to observe the accumulation of polyubiquitinated forms. Total cell extracts were immunoprecipitated with anti-Claudin-1 antibody, followed by immunoblot analysis for ubiquitin. As shown in Figure 4B, RBFOX3 overexpression substantially decreased Claudin-1 ubiquitination. In addition, we did not detect Myc-tagged RBFOX3 in the Claudin-1 immunoprecipitate, which suggests that RBFOX3 does not bind directly to Claudin-1. Overall, our results demonstrated that RBFOX3 increases the stability of Claudin-1 via regulation of the UPP, which is a novel function of cytoplasmic RBFOX3.

The UPP plays critical roles in regulating protein modification and turn-over. This pathway is also important for NF-κB signaling. Phosphorylated IκB, which is induced by various cellular signals, is a target of the HECT-domain E3 ligase for polyubiquitination and subsequent degradation by the proteasome. This releases NF-κB, which translocates inside the nucleus and acts as a transcription factor [21]. We observed that RBFOX3 regulates Claudin-1 levels without direct physical interaction. Therefore, to determine whether RBFOX3 has a role in general protein ubiquitination, we examined NF-κB activity using the NF-κB binding element in the luciferase reporter assay. NF-κB activity decreased with increase in RBFOX3 expression, suggesting that the RBFOX3-mediated decrease in ubiquitination of IκB reduced NF-κB activity (Figure 4C). Therefore, we speculate that RBFOX3 regulates protein stability through attenuation of ubiquitination.

## Discussion

In the present study, we found that *CLDN1* is highly expressed in RBFOX3-positive cells of human lung tissues where localization of RBFOX3 is specifically cytoplasmic. Although RBFOX3 is detected in the cytoplasm of NSCLC or neuroendocrine carcinoma cells [8–10], the molecular function of cytoplasmic RBFOX3 is not studied yet. Here, we discovered the function of cytoplasmic RBFOX3, which is a regulator of Claudin-1 protein stability through attenuation of the latter's ubiquitination.

We observed differences between the intracellular localization of RBFOX3 in the primary lung tissue and RBFOX3-transfected cells. We speculate that the cytoplasmic localization of RBFOX is caused by different splicing isoforms. Alternative splicing of the *Rbfox3* pre-mRNA leads to the production of several protein isoforms, and one of these splicing selections regulates the intracellular localization of RBFOX3 by the inclusion or exclusion of a short C-terminal extension containing the second half of a bipartite hydrophobic proline–tyrosine nuclear localization signal [22]. Therefore, we intend to further investigate the cytoplasmic RBFOX3 variant isoforms in human lung tissue.

RBFOX3 increases the stability of Claudin-1 protein via regulation of the latter’s ubiquitination, without directly interacting with it. Claudin-1 is ubiquitinated by the E3 ubiquitin ligase LNX1p80 in MDCK cells. LNX1 has two splicing variants: LNX1p70 and LNX1p80. LNX1p70 has the N-terminal Numb-binding region and four PDZ domains, whereas LNX1p80 has an additional RING-finger domain related to E3 ubiquitin ligase [18]. However, mRNA expression level of *LNX1p80* was not altered by RBFOX3 expression (results not shown). UPP plays an important role in the maintenance of tissue-specific protein degradation, further identification of molecular mechanism of RBFOX3-mediated ubiquitination will allow us to unveil novel mechanisms of gene expression in controling lung cell function.

RBFOX3, expressed mainly in central nervous system, regulates the generation of alternatively spliced isoforms, which participate in neuronal differentiation [3,23]. In addition, RBFOX3 mutation in humans causes neurodevelopmental delay, cognitive impairments, autistic features, and epilepsy [24]. RBFOX3 plays a role in TGF-β1-induced EMT via regulation of a subset of EMT-related genes such as E-cadherin and Claudin-1 in A549 cells [10]. Although RBFOX3 is predominantly expressed in neural tissues, it is also expressed in human lung tissues [10]. Airway epithelial cells orchestrate functions such as exchange of oxygen and carbon dioxide, protection from foreign substances, and secretion of exocrine and endocrine factors. Therefore, tight junction proteins, including Claudin-1 in lung epithelial cells, are greatly important. As cytoplasmic RBFOX3 expression has relevance with expression of Claudin-1 protein, we speculate that RBFOX3 plays a crucial role in tight junction of lung tissues through regulation of Claudin-1.

Mechanisms of RNA splicing by RBFOX3 have been studied extensively. However, studies on cell type-specific functions of RBFOX3 are rare. Our observations on the cytoplasmic function of RBFOX3 in Claudin-1 expression through ubiquitination regulation might contribute to our understanding of the function of RBFOX3 in human lung tissues.

## Abbreviations

CHX: cycloheximide
EMT: epithelial-mesenchymal transition
GAPDH: glyceraldehyde 3-phosphate dehydrogenase
HDAC: histone deacetylase
HECT: homologous to the E6AP carboxyl terminus
NF-kB: nuclear factor kappa-light-chain-enhancer of activated B cells
NP-40: nonidet P-40
NSCLC: non-small cell lung cancer
RBFOX: RNA binding protein fox-1 (C. elegans) homolog
TGF: transforming growth factor
UPP: ubiquitin–proteasome pathway
